# Electroacupuncture Ameliorates Acute Lung Injury through Promoting Gastrointestinal Motility in Rats with Acute Pancreatitis

**DOI:** 10.1155/2014/943596

**Published:** 2014-04-27

**Authors:** Hui Guo, Shi-Feng Zhu, Rong-Rong Zhang, Xian-Lin Zhao, Mei-Hua Wan, Wen-Fu Tang

**Affiliations:** ^1^Department of Integrative Medicine, West China Hospital, Sichuan University, Chengdu, Sichuan Province 610041, China; ^2^The Second Affiliated Hospital of Shaanxi University of Chinese Medicine, Xi'an, Shanxi Province 712000, China

## Abstract

*Objective*. Gastrointestinal disfunction and acute lung injury (ALI) were common in acute pancreatitis (AP). The effect of electro-acupuncture (EA) on gastrointestinal motility and ALI in rats with AP was investigated to verify the theory of “lung and large intestine are interior exteriorly related” in traditional Chinese medicine. *Methods*. Male Sprague-Dawley rats were randomly divided into the normal group, model group, and EA group. AP model was established by three injections of 20% L-arginine at 1 h intervals. EA were applied to bilateral ST-25 and ST-36 for 30 minutes twice a day after modeling for 3 days. Arterial blood, pancreas, lung, and intestinal tissues were collected for detecting the inflammatory factors and histopathology. Intestinal propulsion rate (IPR) was also measured at 72 h. *Results*. EA treatment improved IPR and increased CCK-8 level compared with model group (*P* < 0.05). It lowered the serum levels of TNF-**α** and IL-6 and increased the level of IL-4 with no effect on IL-10. EA treatment reduced serum vasoactive intestinal peptide (VIP) and myeloperoxidase (MPO) level in the lung and the pathologic scores of pancreas, lung and intestine were decreased (*P* < 0.05). *Conclusion*. EA treatment could promote gastrointestinal motility through inhibiting VIP, and promoting CCK expression and regulate pro- and anti-inflammatory mediators to ameliorate ALI in AP.

## 1. Introduction 

Acute pancreatitis (AP) is the inflammation of the pancreas with high morbidity and mortality. The overproduction of cytokines [1] and inflammatory mediators may account for systemic inflammatory response once the onset of disease of AP, which might cause multiple organ dysfunctions and/or failures if the inflammatory response was out of control, including the gastrointestinal dysfunction and acute lung injury (ALI) at the early stage [2]. Gastrointestinal dysfunction in AP, the trigger of multiple organ failure [1], was related to the intra-abdominal hypertension (IAH) and/or abdominal compartment syndrome (ACS). This commonly leads to ALI or acute respiratory distress syndrome (ARDS), which is difficult to manage up to date with high morbidity and mortality [3, 4]. The approaches to decrease IAH, sustain the intestinal barrier and gastrointestinal function would help inhibit the inflammatory response, enhance the blood SpO2, and improve the clinical results [5]. The optional decompression of ACS in a porcine model of AP incorporating IAH/ACS was associated with significantly reduced mortality, improved systemic hemodynamics, and organ function, as well as alleviated histologic injury and inflammatory intensity of the intestine and lung [6]. So, the decompression of IAH and the control of inflammation would ameliorate the severity of ALI in AP. Unfortunately, there is no specific treatment to control the inflammation and decease the IAH in AP except the invasive surgical decompression all over the world until today with high morbidity and mortality [6].

Traditional Chinese medicine has been adapted for AP for more than 30 years, including Chinese herbal formula and acupuncture. We found that the modified Chinese herbal formula of Dachengqi decoction (DCQD) could relieve IAH and increase the oxygenation index significantly with shorter length of hospital stay for patients with severe AP [7]. Furthermore, DCQD could ameliorate ALI through decreasing IAH and inhibiting the inflammatory response in rats with AP [8]. As well as herbal formula, the effect of acupuncture on the inflammatory response and gastrointestinal motility was also explored. The electroacupuncture (EA) treatment could ameliorate the intestinal paralysis in patients with severe AP [9] and regulate the pro- and anti-inflammatory cytokines in rats with AP [10]. It was found that acupuncture could remarkably reduce the severity of ALI in rats with AP in the acute phase through suppressing the overexpression of serum macrophage inflammatory protein-2 (MIP-2) mRNA in the lung and large intestine tissues, lowering the level of serum MIP-2 [11].

Along these studies, we know that acupuncture could regulate the inflammatory response, promote the gastrointestinal motility, and ameliorate the lung injury in AP. However, it is still unclear whether acupuncture could ameliorate ALI through promoting gastrointestinal function and related inflammation based on the theory in traditional Chinese medicine of “Lung and Large intestinal exterior-interiorly related.” The present study aimed to explore the effect of EA on ALI through regulating the gastrointestinal dysfunction and inflammatory response in rats with AP.

## 2. Material and Methods

### 2.1. Animal Experiment

Eighteen male SD rats of 160–200 g, 8–10 weeks old and clearing were obtained from the animal center of West China hospital of Sichuan University. They were randomly divided into normal group, model group, and EA group. The animal study was performed according to the Guide for the Care and Use of Laboratory Animals of the National Institutes of Health. The protocol was approved by the Ethics Committee for Animal Experiments of our hospital. Rats were intraperitoneally injected with 20% L-arginine (100 mg/100 g) at 1 h intervals for three times to induce AP [12].

### 2.2. EA Treatment Protocol

EA group rats were binding against slipping and turning. Bilateral ST-25 and ST-36 were pierced with acupuncture needles of type 32, 0.23 mm∗13 mm, and stimulated by SDZ-II-type EA treatment instrument (2 Hz/100 Hz, 2 mA) for 30 minutes twice a day after modeling for 2 days [13, 14]. Normal control group and model group were binding for the same time.

### 2.3. Sample Collection and Intestinal Propulsion Rate Measurement

Phenolsulfonphthalein 0.5 mL (2 mg/mL) was dosed to rats by intragastric infusion 30 min before scarification. Intestinal propulsion rate (IPR) was identified as ratio of the phenolsulfonphthalein promoting distance and the total length of the small intestine [15]. Rats were sacrificed 72 h after modeling and blood was obtained from heart. Atrial blood, pancreas, lung, and intestinal tissues were dissected immediately and collected for biomarkers and histopathology.

### 2.4. Serum and Tissue Measurement

Blood was centrifuged at 3000 rpm for 15 min and the serum was stored at −20°C. Serum CCK, VIP, TNF-*α*, IL-4, IL-6, and IL-10 were determined by enzyme-linked immunosorbent assay (ELISA, Kits from Nanjing Jiancheng Bioengineering Institute). As previously described, the accumulation of neutrophils in the lungs was assessed by determination of myeloperoxidase (MPO) activity [16]. Briefly, the frozen tissue samples were thawed and suspended in 10% phosphate buffer (pH 6.0) containing 1% hexadecyltrimethylammonium bromide. The samples were sonicated on ice and centrifuged at 12000 rpm for 15 min at 4°C. An aliquot (30 *μ*L) was transferred into 180 *μ*L of phosphate buffer (pH6.0) containing 0.167 mg/mL o-dianisidine dihydrochloride and 0.0005% hydrogen peroxide (Sigma-Aldrich). The change in absorbance was read at 490 nm.

### 2.5. Pathological Assessment of Tissues

Parts of the tissues were fixed and embedded in paraffin wax for histological analysis. Pancreas, lung, and small intestine were scored by an experienced pathologist from West China Hospital of Sichuan University in a blinded fashion. The pathological scoring standard of pancreas includes edema, bleeding, inflammatory cell infiltration, and necrosis according to Schmidt's report [17].

### 2.6. Statistics

Statistical analysis was performed with the PEMS3.1 for Windows (Sichuan University, China). Data are expressed as mean ± SEM. Statistical analysis was performed using one-way analysis of variance followed by Dunnett's test for each paired experiment value of *P* < 0.05 which was considered to be significant.

## 3. Results

### 3.1. Electroacupuncture Treatment Regulates Acute Pancreatitis-Induced Serum CCK and VIP and Improves Intestinal Propulsion

**Figure 1:**
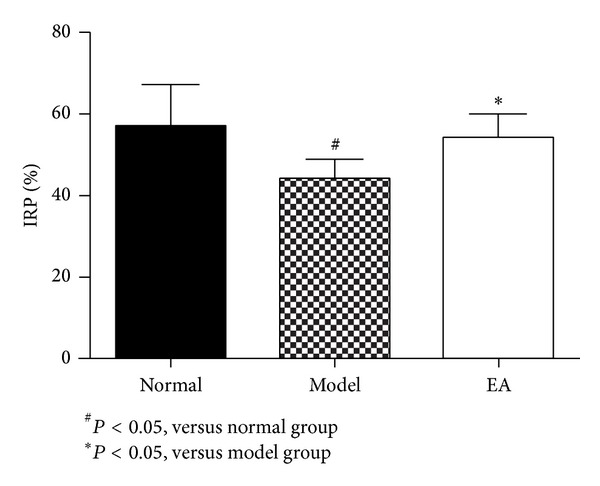
Comparison of intestinal propulsion rate (IRP) (*n* = 6).

As shown in Figure 1, IPR in model group was significantly slower than that in normal rats. After EA treatment, IPR in EA group was much faster than that in the model group (Figure 1). IPR in EA group and normal group was similar. This demonstrated that EA management could restore the intestinal motility affected in the AP.

### 3.2. EA Treatment Decreases Serum Level of CCK-8 and Increases VIP Level

**Figure 2:**
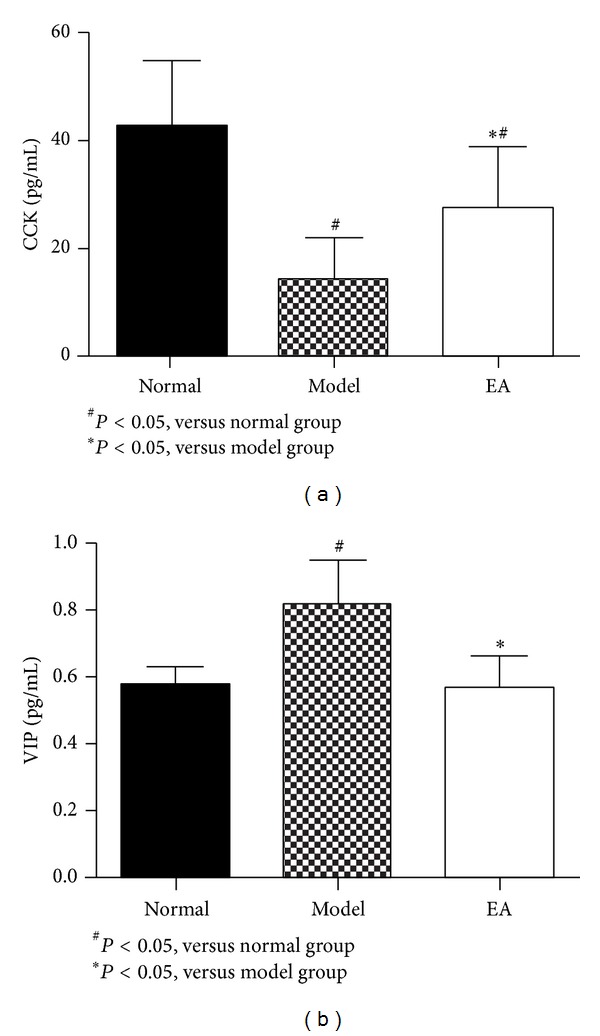
Comparison of serum CCK and VIP among rats of the three groups (mean ± SD) pg/mL.

As shown in Figure 2, the serum level of CCK in AP 72 h after modeling was obviously lower than that in normal rats. After 2 days treatment, CCK in EA group increased significantly, but it is still lower than that in the normal group, which meant that EA treatment could increase the serum level of CCK. To the contrary, the serum level of VIP in L-arginine induced AP rates was much higher than that in normal group. Compared to the model rats, 2 days of EA treatment significantly lowered the serum VIP (Figure 2). All these showed that EA could promote the intestinal motility through regulating the expression of CCK and VIP.

### 3.3. EA Treatment Attenuates Acute Pancreatitis-Induced Increases in Serum TNF-*α*, IL-6 and Increases IL-4

**Figure 3:**
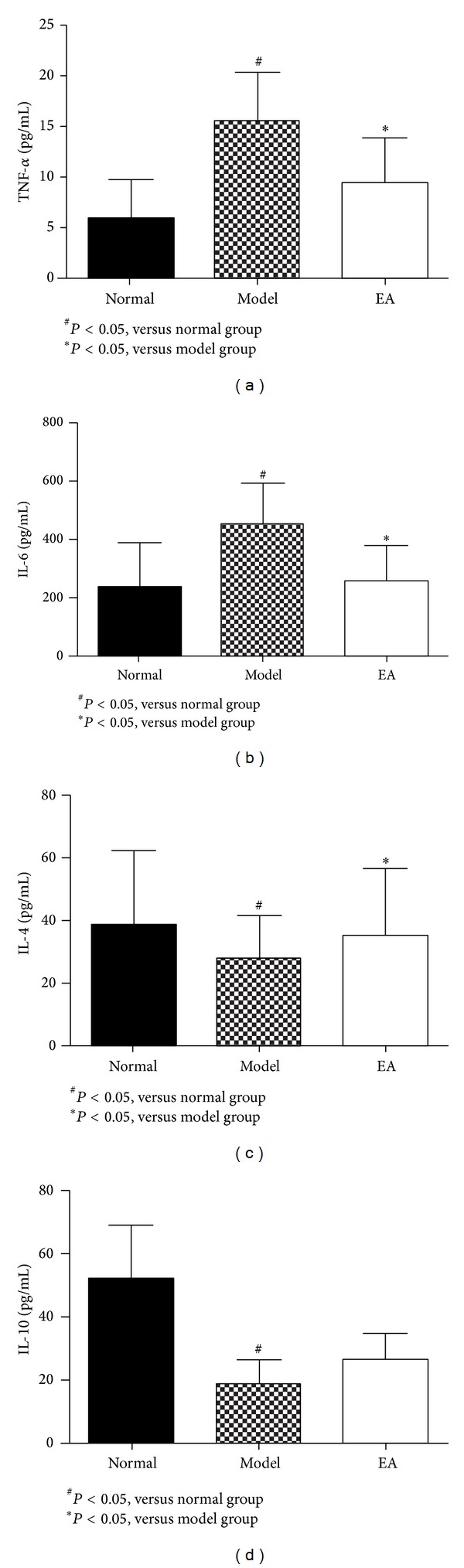
Comparison of serum TNF-*α*, IL-6, IL-4, and IL-10 among groups (mean ± SD) pg/mL.

As shown in Figure 3, serum levels of proinflammatory TNF-*α* and IL-6 were increased (Figures 3(a) and 3(b)) and anti-inflammatory IL-4 and IL-10 were decreased after induction of AP in rates (Figures 3(c) and 3(d)). Compared to the model rats, EA could decrease the serum levels of TNF-*α* and IL-6 significantly after 2 days of treatment (Figures 3(a) and 3(b)). EA treatment could also increase IL-4 levels in rates with SAP significantly (Figure 3(c)), with no effect on IL-10 (Figure 3(d)). All these data displayed that EA treatment could regulate the inflammatory response in rats with AP via inhibiting the proinflammatory mediators and promoting the anti-inflammatory mediator.

### 3.4. EA Treatment Ameliorates Acute Pancreatitis-Induced Changes in Histopathology and MPO

**Figure 4:**
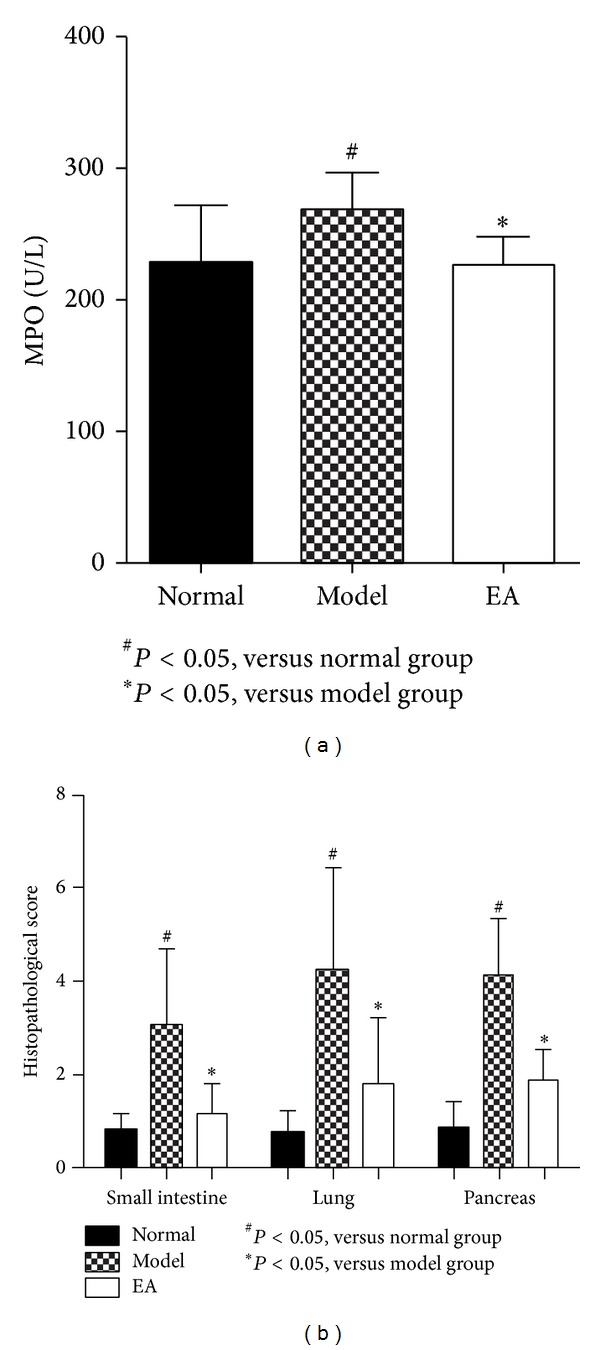
MPO in lung (a), pathological changes of pancreas, lung, and small intestine (b).

As shown in Figure 4(a), MPO activities in the lung of AP model group were markedly higher than those in the normal group. The level of MPO in EA group was significantly lower than that in the model group after 2-day treatment in rats with AP. This showed that EA treatment could ameliorate ALI induced by AP, which is further identified by the pathological assessment.

**Figure 5:**
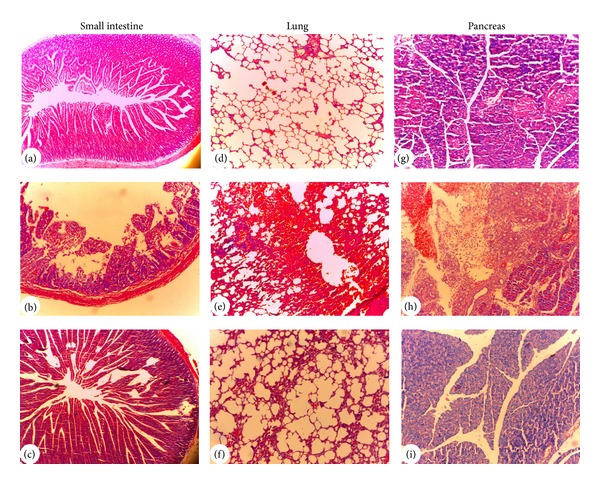
Histopathological findings of pancreatic, lung, and small intestinal tissues observed by hematoxylin and eosin staining (pancreatic and lung tissues: light microscopy, ×100; small intestinal tissue: light microscopy, ×40): in the photos of small intestinal tissue, the damage of intestinal mucosa was significant, in the photos of lung tissue, pulmonary interstitial edema and infiltration of large quantity of inflammatory cells were found in the model group, while the situation was much lighter in the EA group; and in the photos of pancreatic tissue, infiltration of large quantity of inflammatory cells was found in the model group, while the situation was much lighter in the EA group.

Administration of intraperitoneally injections L-arginineat 1 h intervals for three times showed features of typical AP characterized by unclear pancreas acinar structure caused by moderate to severe interstitial edema, extensive inflammatory cell infiltration, parenchymal necrosis, and hemorrhage (Figure 5(h)) [12]. There were also marked pulmonary interstitial edema and inflammatory infiltration with alveolar collapse in the lungs (Figure 5(e)), which was similar to Dawra's research [18]. Moreover, the damage of intestinal mucosa was obvious, while the intestinal structural is relatively complete in the EA group (Figure 5(b)). After EA treatment, three groups of pathologic score ofpancreatic, lung, and intestinal tissues were much lower than model group (Figure 4(b)). All these data showed that 2-day EA treatment could improve the tissue pathological insult of intestine, lung, and at last pancreas.

 In Figure 5: (a), (b), and (c) represent small intestine; (d), (e), and (f) represent lung tissue; (g), (h), and (i) represent pancreas tissue; (a), (d), and (g) represent normal group; (b), (e), (h) represent model group; (c), (f), and (i) represent EA group.

## 4. Discussion and Conclusion

The present study found that EA treatment could promote the intestinal propulsion rate through regulating the expression of CCK and VIP, decreasing the serum levels of TNF-*α* and IL-6, and increasing IL-4 levels in rates with SAP significantly after 2 days of treatment (Figures 3(a) and 3(b)). At the same time, two-day EA treatment could improve the tissue pathological injury of intestine, lung, and at last pancreas. It was concluded that EA could promote the intestinal motility and regulate the related inflammatory response, which lead to the amelioration of lung injury in rats with SAP.

Gastrointestinal dysfunction, the common clinical symptom in acute pancreatitis, can cause IAH and be the trigger of multiple organ failures [3, 4]. The first injured organ of lung commonly demonstrated with acute respiratory distress syndrome (ARDS), which is a typical index of the revised Atlanta classification for SAP. It is important to prevent the occurrence of ARDS in the early stage of acute pancreatitis, including the management of IAH and ACS with gastrointestinal dysfunction. In this study, the EA treatment was used to promote the intestinal motility, decrease IAH, and then ameliorate the related lung injury in SAP based on the theory of “lung and large intestine are interior exteriorly related” in traditional Chinese medicine. First, we identified that EA could promote the intestinal motility by increasing the IPR and regulating the expression of CCK and VIP. Recent studies found that disorder of gastrointestinal hormone such as CCK, VIP, and motilin (MTL) played an important role in gastrointestinal dysfunction [16, 19]. Blood CCK mainly comes from the intestinal secretory endothelial cells, promoting the contraction of the gallbladder and relaxation of the sphincter of Oddi and protecting the gastric mucosa [20]. All these showed that EA could promote the gastrointestinal motility via regulating the expression of related hormones.

Second, the injured intestinal tissue evoke and worsen the inflammatory response in SAP, including the bacterial translocation. The levels of proinflammatory mediators are elevated in the course of acute pancreatitis and are involved in the inflammatory cascade reaction to the pancreatic acinar cell damage, including TNF-*α*, IL-1, and IL-6 [21]. In this study, we found that EA increase the expression of IL-4 and inhibit the expression of TNF-*α* and IL-6 as well as increase the IPR in rats with SAP, which displayed that EA might regulate the inflammatory response while it promoted the intestinal motility. Former study reported that EA at ST 36 could downregulate the serums TNF-*α* and IL-6 in rates with sodium taurocholate-induced acute pancreatitis [22], which is similar to our results. All these showed that EA could inhibit the related inflammatory response. On the other hand, our study found that EA treatment might increase the level of anti-inflammatory cytokines IL-4 to relieve inflammation response in rats with acute pancreatitis. This is similar to the study that EA treatment of Zusanli (ST-36), Shangjuxu (ST-37), Quchi (LI-11), and other points could reduce the expression of IL-1*β* in intestine and elevate the serum level of IL-4 in rats with ulcerative colitis [23], which demonstrated that EA could promote the expression of anti-inflammatory mediators. It was deduced that EA could promote the intestinal motility through regulating the related inflammatory response in gastrointestinal.

Regarding IL-10, Pezzilli et al. and Myer et al. showed that IL-10 concentrations in SAP patients were significantly higher than those in mild AP. In this study, we found no difference in IL-10 concentration between EA group and model group rates. This is contrary to the result that acupuncture Tianshu (ST-25) point could increase the serum level of IL-10 in rats with sodium taurocholate-induced SAP [10]. This may be due to the different modeling method with sodium taurocholate or 20% L-arginine (100 mg/100 g) at 1 h intervals for three times, which lead to different disease severity of acute pancreatitis.

In addition, CCK-8 can inhibit the increase of TNF-*α*, IL-1, IL-6, and other proinflammatory cytokines [24, 25]. Xu et al. found that serums MTL and CCK decreased while VIP increased significantly in acute pancreatitis patients [16]. Wang et al. have found that EA treatment could increase colonic transit time (CTT) and serum CCK-8 and decrease serum VIP [26]. In the present study, EA treatment could increase serum CCK-8 concentrations and decrease serum VIP concentration significantly to regulate intestinal propulsion in L-arginine induced acute pancreatitis rates. All those showed that disorders of gastrointestinal hormone and related inflammatory response were partly on account of gastrointestinal dysfunction in acute pancreatitis and acupuncture is able to enhance the gastrointestinal dynamics and improve its motor activity through regulating the gastrointestinal hormone and their related inflammatory response mediators.

Third, the histopathology of lung was improved when EA promoted the intestinal motility and regulated the inflammatory response, with the decrease of MPO in the lung of AP and the pathological amelioration of lung. It was concluded that EA might ameliorate the lung injury via inhibiting the intestinal inflammatory response and promoting the gastrointestinal motility. Paralysis of intestine can aggravate the intestinal barrier resulting in “bacterial translocation” and “endogenous intestinal endotoxemia” and cause a “second strike” on body [27, 28]. The worse the intestinal dysfunction was, the more the neutrophil accumulated in the lung and the higher the levels of MPO increased, which displayed that the lung injury in the course of acute pancreatitis is associated with the motility and inflammatory response of the gut [29]. Our previous study found that improving gastrointestinal motility could relieve the IAH and reduce water content and MPO of the lung in rats with acute pancreatitis [8]. Other studies have suggested that acupuncture Zusanli (ST-36) could significantly reduce intestinal permeability in patients with acute pancreatitis and decrease endogenous inflammatory mediators and vasoactive substances in the intestinal mucosa membrane to ameliorate the intestinal epithelial cell necrosis for protecting gastrointestinal mucosa barrier [30]. This is similar to the theory in traditional Chinese medicine of “Lung and Large intestinal exterior-interiorly related.” In the present study, our results indicated that EA treatment promotes gastrointestinal propulsion and ameliorates acute pancreatitis-induced intestinal histopathology, which might finally relieve the lung injury in SAP.

What is more, studies have found that CCK-8 could stimulate the tracheal respiration, make the trachea relaxed, and relieve the endotoxemia in rats with pulmonary hypertension. CCK-8 could also restrain the in vitro lipopolysaccharide (LPS) and activate pulmonary interstitial macrophages (PIM) to reduce the endotoxemia related inflammatory changes in the lung [31]. VIP, a straight-chain peptides, is widely distributed in the gastrointestinal tract, lung, and intestinal tract which mainly promote glandular secretion. It also has a negative relationship with gastrointestinal movement. The present study found that EA could promote intestinal motility through downregulation of VIP [32]. On the other hand, VIP is a vasodilator effect of vasoactive compounds, which has potent pulmonary vascular expansion and inhibits pulmonary artery smooth muscle cell hyperplastic biological activity [33]. It has been demonstrated in animal experiments that VIP could reduce the effect of pulmonary vascular resistance to decrease the pulmonary hypertension [34, 35]. VIP has a similar effect on expansion pulmonary vascular resistance in nitric oxide (NO) and activates adenylate cyclase to relax pulmonary artery smooth muscle cell [36]. It showed that CCK and VIP had some protective effects on the lung. In this study, serum VIP in EA group was lower than that in the model group, which suggested that acupuncture may facilitate the recovery of gastrointestinal function and relieve the lung injury through reducing the serum level of VIP.

In summary, the present findings demonstrated that EA treatment may be capable of attenuating the severity of acute pancreatitis and associated lung injury in rates. The potential mechanism might be that EA treatment improves the gastrointestinal dysfunction through regulating gastrointestinal hormone and related inflammatory mediators, which is in accord with the theory of “Lung and Large intestinal exterior-interiorly related.”

## Abbreviations

AP: Acute pancreatitis
EA: Electro-acupuncture
ALI: Acute lung injury
IPR: Intestinal propulsion rate
VIP: Vasoactive intestinal peptide
MPO: Myeloperoxidase
IAH: Intra-abdominal hypertension
ACS: Abdominal compartment syndrome
ARDS: Acute respiratory distress syndrome
DCQD: Dachengqi decoction.

## References

[1] Zhang C, Ge C-L, Guo R-X, He S-G (2005). Effect of IL-4 on altered expression of complement activation regulators in rat pancreatic cells during severe acute pancreatitis. *World Journal of Gastroenterology*.

[2] Pooran N, Indaram A, Singh P, Bank S (2003). Cytokines (IL-6, IL-8, TNF): early and reliable predictors of severe acute pancreatitis. *Journal of Clinical Gastroenterology*.

[3] Pelosi P, Quintel M, Malbrain ML (2007). Effect of intra-abdominal pressure on respiratory mechanics. *Acta Clinica Belgica*.

[4] de Waele JJ, Hesse UJ (2005). Life saving abdominal decompression in a patient with severe acute pancreatitis. *Acta Chirurgica Belgica*.

[5] Zhao G, Zhang JG, Wu HS (2013). Effects of different resuscitation fluid on severe acute pancreatitis. *World Journal of Gastroenterology*.

[6] Ke L, Ni HB, Tong ZH, Li WQ, Li N, Li JS (2013). The importance of timing of decompression in severe acute pancreatitis combined with abdominal compartment syndrome. *Journal of Trauma and Acute Care Surgery*.

[7] Wan MH, Li J, Huang W (2012). Modified Da-Cheng-Qi decoction reduces intra-abdominal hypertension in severe acute pancreatitis: a pilot study. *Chinese Medical Journal*.

[8] Wan M-H, Li J, Tang W-F (2011). The influnence of Dachengqi tang on acute lung injury and intra abdominal hypertension in rats with acute pancreatits. *Journal of Sichuan University*.

[9] Luo Y-H, Zhong G-W, Zhao S-P, Tang H-M, Zhang L-N (2011). Efficacy observation of electroacupuncture intervention on severe acute pancreatitis at early stage complicated with intestinal paralysis. *Zhongguo Zhen Jiu*.

[10] Xue Q-M, Huang L, Li N (2011). Effects of electroacupuncture at Tianshu (ST25) on pro- and anti-inflammatory cytokines in rats with severe acute pancreatitis. *Zhong Xi Yi Jie He Xue Bao*.

[11] Jiang LY, Huang JR, Zhao HQ, Zhu JF, Dai JL, Zhang WD (2013). Effect of acupuncture on serum MIP-2 and MIP-2 mRNA expressions in isolated Fei and Dachang of severe acute pancreatitis induced acute lung injury rats in the acute phase. *Zhongguo Zhong Xi Yi Jie He Za Zhi*.

[12] Long YM, Chen K, Xie WR (2006). Establish of rat model of mild acute pancreatits with intraperitoneal injection of L-arginine. *Journal Guangdong College of Pharmacy*.

[13] Li ZR (2004). *Experimental Acupuncture Science*.

[14] Wang ZJ, Li WM (2010). Regulation of abnormal electricity for irritable boewl syndrome intestinal movement. *Journal of Chinese Integrative Medicine*.

[15] Wu YM, Wang P, Wei MX (2010). Effect of different concentration of licorice on gastrointestinal motion of mice. *Jiangsu Journal of Traditional Chinese Medeicine*.

[16] Xu M, Wang XP, Yuan YZ (2002). Gastrointestinal dysmotility in patients with acute pancreatitis. *Chinese Journal of Emergency Medicine*.

[17] Schmidt J, Rattner DW, Lewandrowski K (1992). A better model of acute pancreatitis for evaluating therapy. *Annals of Surgery*.

[18] Dawra R, Sharif R, Phillips P, Dudeja V, Dhaulakhandi D, Saluja AK (2007). Development of a new mouse model of acute pancreatitis induced by administration of L-arginine. *American Journal of Physiology: Gastrointestinal and Liver Physiology*.

[19] Cote F, Pare P, Friede J (1995). Physiological effect of cholecystokinin on gastric emptying of liquid in functional dyspepsia. *American Journal of Gastroenterology*.

[20] Zhang X, Han D, Ding D (2002). Cholecystokinin octapeptide inhibits the in vitro expression of CD14 in rat pulmonary interstitial macrophages induced by lipopolysaccharide. *Chinese Medical Journal*.

[21] Bhatia M, Neoptolemos JP, Slavin J (2001). Inflammatory mediators as therapeutic targets in acute pancreatitis. *Current Opinion in Investigational Drugs*.

[22] Xue Q-M, Ning L, Xue P, Wang C-W, He H-B (2011). Effect of electroacupuncture on serum proinflammatory cytokine Levels and pancreatitic nuclear factor Kappa-B expression in acute pancreatitis rats. *Acupuncture Research *.

[23] Yang QY, Zhang H, Xiang ZY (2008). Effect on electroacupuncture on serum IL-1*β* and IL-4. *World Health Digest*.

[24] Li SJ, LingYL, Wang DH (2001). Effect of cholecystokinin octapeptide on change in rabbit thoracic aortic reactivitis induced by lipopolysaccharides in vivo. *Chinese Journal of Pathophysiology*.

[25] Ling Y-L, Meng A-H, Zhao X-Y, Shan B-E, Zhang J-L, Zhang X-P (2001). Effect of cholecystokinin on cytokines during endotoxic shock in rats. *World Journal of Gastroenterology*.

[26] Wang X-Y, Shi X, He L (2007). Effect of electroacupuncture on gastrointestinal dynamics in acute pancreatitis patients and its mechanism. *Current Research Acupuncture*.

[27] Yang SL (2002). Research on Exterior-interior relation between lung and large intestine. *Chinese Journal of Integrated Traditional and Weatern Medicine on Digestion*.

[28] Ogawa M (1998). Acute pancreatitis and cytokines: “Second attack” by septic complication leads to organ failure. *Pancreas*.

[29] Chen P, Wang WW, Wang G (2010). An experimental study on protective effect of carbon monoxide releasing molecule on severe acute pancreatitis induced lung injury. *Chinese Journal of Hepatobiliary Surgery*.

[30] Wang X-Y (2007). Electroacupuncture for treatment of acute pancreatitis and its effect on the intestinal permeability of the patient. *Chinese Acupuncture and Moxibustion*.

[31] Gao W-J, Xu S-J, Cong B, Li S-J, Ma C-L (2007). Activation of cAMP-PKA signal pathway by CCK-8 in rat pulmonary interstitial macrophages. *Chinese Pharmacological Bulletin*.

[32] Zha BG, Liu M, Wang ZY (2008). Effect of “ Wumo Plaster” on gastrointestinal function and vasoactive intestinal peptide in patients undergoing abdominal operation. *Shanghai Journal of Traditional Chinese Medicine*.

[33] Jeffery TK, Morrell NW (2002). Molecular and cellular basis of pulmonary vascular remodeling in pulmonary hypertension. *Progress in Cardiovascular Diseases*.

[34] Newman JH (2005). Pulmonary hypertension. *American Journal of Respiratory and Critical Care Medicine*.

[35] Runo JR, Loyd JE (2003). Primary pulmonary hypertension. *The Lancet*.

[36] Topper JN (2000). TGF-*β* in the cardiovascular system: molecular mechanisms of a context-specific growth factor. *Trends in Cardiovascular Medicine*.

